# Testing for the Presence of Correlation Changes in a Multivariate Time Series: A Permutation Based Approach

**DOI:** 10.1038/s41598-017-19067-2

**Published:** 2018-01-15

**Authors:** Jedelyn Cabrieto, Francis Tuerlinckx, Peter Kuppens, Borbála Hunyadi, Eva Ceulemans

**Affiliations:** 1 Research Group of Quantitative Psychology and Individual Differences, KU Leuven–University of Leuven, Leuven, Belgium; 2 Department of Electrical Engineering (ESAT), STADIUS Center for Dynamical Systems, Signal Processing and Data Analytics, KU Leuven–University of Leuven, Leuven, Belgium; 3 Imec, Leuven, Belgium

## Abstract

Detecting abrupt correlation changes in multivariate time series is crucial in many application fields such as signal processing, functional neuroimaging, climate studies, and financial analysis. To detect such changes, several promising correlation change tests exist, but they may suffer from severe loss of power when there is actually more than one change point underlying the data. To deal with this drawback, we propose a permutation based significance test for Kernel Change Point (KCP) detection on the running correlations. Given a requested number of change points *K*, KCP divides the time series into *K* + 1 phases by minimizing the within-phase variance. The new permutation test looks at how the average within-phase variance decreases when *K* increases and compares this to the results for permuted data. The results of an extensive simulation study and applications to several real data sets show that, depending on the setting, the new test performs either at par or better than the state-of-the art significance tests for detecting the presence of correlation changes, implying that its use can be generally recommended.

## Introduction

Detecting correlation changes in multivariate time series is relevant across a wide spectrum of fields^1–4^. Take for instance an important biomedical data processing problem, the detection of epileptic seizures: The onset of seizures is often characterized by excessive synchronization of electrical signals in the brain^5–7^. In functional neuro-imaging, brain correlation networks are expected to alter when an individual has to perform multiple tasks in a row^8^. Climate trends are studied by confirming changes in the connections of important climate indices^9^. Crises in the financial market are typically marked by drastic correlation changes of several stocks^10^.

When the goal is to detect abrupt correlation changes, one can turn to change point detection methods to capture whether and when they exactly occur. In practice, when researchers do not have a strong priori information on the distribution of the data, non-parametric variants of this tool are rather attractive. Aside from general purpose methods (Kernel Change Point Detection (KCP)^11^, Decon^12^, E-divisive^13^ and Multirank^14^) which can pick up other changes such as means and variances next to correlation changes (and therefore have lower power to pick up correlation changes), a number of methods have been proposed that are specifically dedicated to tracing correlation changes. These methods include the Frobenius and Maximum norm procedures^8^ and the Cusum method^15^.

These latter methods handle the detection of correlation changes by locating the most likely correlation change point and then testing whether there is a significant difference in correlations before and after. When the change point is found significant, the same methods scan the identified phases for the next change points. However, when the test to detect the first change point turns out to be non-significant, the methods terminate, leading to the conclusion that the whole time series has a constant correlation. Testing for the presence of a single significant change point, although sensible at first sight, may suffer from a loss of power in cases when there are multiple change points present in the time series, however.

**Figure 1:**
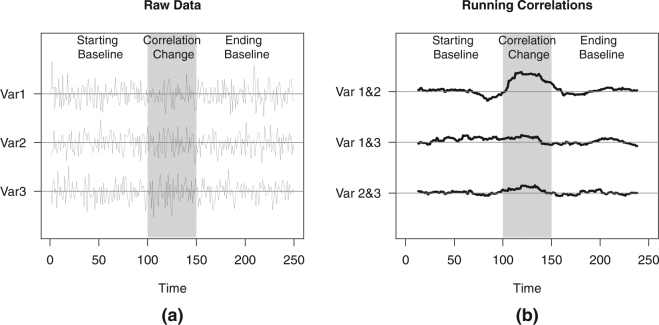
Time series and running correlation plots for the toy example. In (**a**), a time series comprised of three variables drawn from a multivariate normal distribution with zero means is displayed. In the baseline phases (white background), variables are uncorrelated and have a unit variance such that the covariance matrix is equal to $$[\begin{array}{ccc}1 & 0 & 0\\ 0 & 1 & 0\\ 0 & 0 & 1\end{array}]$$. In the middle phase (gray background), an event is introduced such that the correlation between the first two variables increase, giving a covariance matrix of $$[\begin{array}{ccc}1 & 0.7 & 0\\ 0.7 & 1 & 0\\ 0 & 0 & 1\end{array}]$$. In (**b**), the running correlations obtained using a window size of 25 time points are exhibited.

To illustrate this power problem for the Frobenius norm procedure, consider a toy example comprised of three phases, implying two change points (Fig. 1). The first phase is a baseline phase, where three monitored variables are uncorrelated. Then at the onset of the middle phase, an event occurs (indicated by the start of the gray background shading), causing two of them to become highly correlated. Finally, in the third phase, the correlations become zero again, implying a return to the baseline phase. As we will see in the next section, the Frobenius norm procedure locates the most plausible change point at the start of the middle phase. When testing the significance of this change point, the “before change point” phase corresponding to the starting baseline, will then be compared to the “after change point” phase comprised of two underlying phases: the event and return to baseline phase. This pooling of two phases, however, yields a considerably weaker correlation estimate compared to the extreme correlation exhibited by the correlating variables within the bounds of the event. Thus, the one change point based test generates a statistic that does not capture the real magnitude of the abrupt changes in the data, and consequently, yields less power to declare a significant change point. A similar reasoning holds for maximum norm and Cusum as we will show in the next section. Clearly, this behavior is problematic as these tests may fail to signal the presence of a true single correlation change in the data, and therefore the methods could not be applied further, sequentially, to pick up multiple change points.

In this paper, we propose a new permutation based test that takes into account the possibility of multiple change points occurring in the data. The new test will be called the KCP permutation test, as it builds on the best currently available general purpose method, KCP (for an extensive simulation, see Cabrieto, Tuerlinckx, Kuppens, Grassmann and Ceulemans^16^). To focus on the correlation changes, we apply KCP on the running correlations instead of the raw data. Given a requested number of *K* change points, KCP divides the running correlations time series into *K* + 1 phases with an as low within-phase variance as possible. The new permutation test compares the decrease in the average within-phase variance for increasing *K* between the observed and permuted data. Our approach therefore considers several change point solutions simultaneously, in contrast with the sequential procedures above, which only consider a single change point (*K* = 1). Through further analyses of the toy example (in which the different contrasted tests will be more extensively introduced), an extensive simulation study, and three illustrative applications on EEG epileptic seizure detection, psychopathology, and stock returns, we will exhibit that the proposed KCP permutation test performs either at par, or, in many cases, better than recently proposed tests, implying that its use can be generally recommended.

## Results

### Toy Example

With this toy example, we will demonstrate that in case of multiple change points, the KCP permutation test is more powerful than the three single change point based tests: the Frobenius and Maximum norm tests and Cusum. We generated a trivariate time series comprised of three phases, implying two change points. The three variables were drawn from a multivariate normal distribution with zero means and unit variance. As described earlier, the first phase ***X***_1:100_ is a starting baseline, comprised of 100 time points, where the variables are uncorrelated such that the covariance matrix is given by $${{\boldsymbol{\Sigma }}}_{1:100}=[\begin{array}{ccc}1 & 0 & 0\\ 0 & 1 & 0\\ 0 & 0 & 1\end{array}]$$. In the middle phase consisting of 50 time points, ***X***_101:150_, the first two variables are strongly correlated such that the covariance matrix becomes $${{\boldsymbol{\Sigma }}}_{101:150}=[\begin{array}{ccc}1 & 0.7 & 0\\ 0.7 & 1 & 0\\ 0 & 0 & 1\end{array}]$$. The last phase is a return to baseline phase, ***X***_151:250_, comprised of 100 time points, where all the variables became uncorrelated again.

In the next paragraphs, we first describe the results for the single change point based tests. We then introduce the KCP permutation test and demonstrate how it makes use of the multiple change points solutions, yielding more power to detect the presence of at least one change.

### Single Change Point Based Tests

#### Frobenius and maximum norm tests

**Figure 2:**
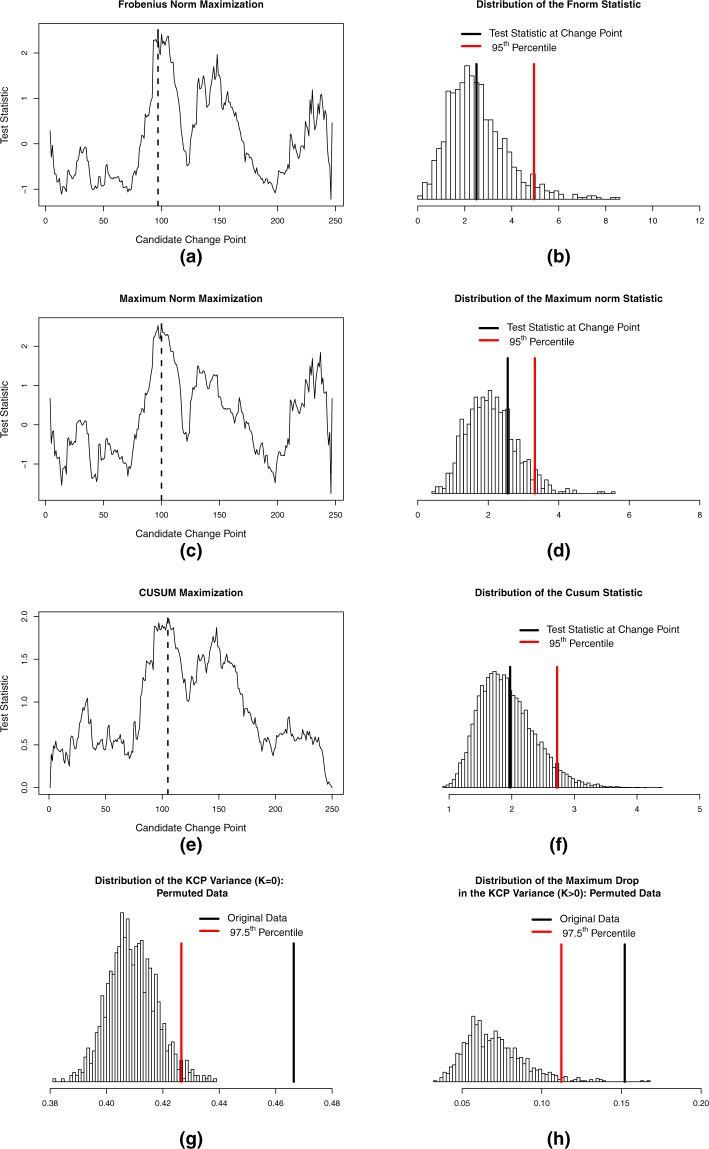
Significance testing to detect the presence of correlation change. In (**a**), (**c**) and (**e**), the maximization of the test statistic to locate the most plausible change point (assuming *K* = 1) for the Frobenius norm, Maximum norm and Cusum tests, respectively, is shown. The maximum is indicated by the broken vertical line and the most plausible change point is the time point after it. The most plausible change points, *T* = 98, and *T* = 106, for the Frobenius norm and Cusum tests, respectively, are proximal to the first change point introduced at *T* = 101, while that of Maximum norm is located exactly at that change point. In (**b**),(**d**) and (**f**), the corresponding significance testing done by comparing the test statistic to the distribution under the null is illustrated. These three tests yielded non-significant results as the obtained test statistics fall in the middle of the reference distributions. For the KCP permutation test, the variance and the variance drop tests are shown in (**g**) and (**h**), respectively. Bonferroni correction was employed to correct for multiple testing, thus each test has an α equal to 0.025. Both tests yield significant results as the variance at *K* = 0 and the maximum variance drop at *K* = 2 for the toy example exceeded the 97.5^th^ percentile cut-off of the corresponding distribution obtained from the permuted data.

Frobenius norm test: The test statistic of the Frobenius norm test equals the squared Frobenius norm of the differences between the correlations before and after the change point. This statistic is thus calculated by squaring and summing all the differences of the correlation matrices before and after the change point. If there is no change point, the differences will be small, and the squared Frobenius norm will be close to zero. However, if there is a correlation change in the data, the differences will be more pronounced. Consequently, the squared Frobenius norm will be large. For the toy example, the squared Frobenius norm and thus the test statistic is maximized if the first phase runs until *T* = 97, implying a change point at *T* = 98, which is just 3 time points before the first real change point at *T* = 101 (see Fig. 2a).

The significance test, however, did not generate a significant result, as the test statistic did not exceed the cut-off for the reference distribution (see Fig. 2b). This demonstrates that for the toy example, the Frobenius norm test did not have the required power to declare the correlation change significant (despite the obvious change). We attribute this weakness to the fact that the Frobenius norm test imposes the comparison of two phases only. The maximal test statistic was obtained when computing the differences between the correlation matrices for ***X***_1:97_ and ***X***_98:250_, the generated before and after phases, respectively. Whereas the correlation estimates, $${\hat{{\boldsymbol{\Sigma }}}}_{1:97}=[\begin{array}{ccc}1 & -0.06 & 0.14\\ -0.06 & 1 & 0.00\\ 0.14 & 0.00 & 1\end{array}]$$ of the before-change point phase strongly resemble the underlying baseline phase, ***X***_1:100_, the after-change point correlation estimates, $${\hat{{\boldsymbol{\Sigma }}}}_{98:250}=[\begin{array}{ccc}1 & 0.31 & 0.09\\ 0.31 & 1 & 0.14\\ 0.09 & 0.14 & 1\end{array}]$$, were obtained by pooling the time points from two underlying phases: the event and the return to baseline phase. Thus, although we introduced a strong correlation change of 0.7 for the first two variables during the event, the correlation difference for this pair contributing to the Frobenius norm computation equals

$${\hat{{\boldsymbol{\Sigma }}}}_{1:97}[1,2]-{\hat{{\boldsymbol{\Sigma }}}}_{98:250}[1,2]=-0.06-0.31=-0.37,$$

which is considerably smaller. All other differences are expected to be small, because no other changes were induced. Note that if the last 100 time points (the 3^rd^ phase) are discarded, such that there is only one change point at *T* = 101, the Frobenius norm test indeed yields a significant result.

Maximum norm test: Another weakness of Frobenius norm, which is also the case for the toy example, is that when there is only a subset of variables changing, the test statistic may not increase dramatically since it also includes other non-changing variable pairs in the computation. To better detect changes occurring in a small subset of variables, Barnett and Onnela^8^ proposed the maximum norm test, which only looks at the maximum absolute difference between the before and after correlations and disregards the rest of the elements in the difference matrix. For the toy example, the most plausible change point according to the maximum norm is located at *T* = 101 (see Fig. 2c), which is exactly the first underlying change point. However, the test is still non-significant (see Fig. 2d), due to the pooling of the last two phases when calculating the correlations.

#### Cusum test

The Cusum test is based on the idea that if there is no correlation change, the maximum sum of the absolute differences between the overall correlations (computed using all observations in the time series, ***X***_1:*n*_) and the correlations up to time point *i*, (computed using only the observations, ***X***_1:*i*_ with *i* varying between 2 and *n*), should fluctuate as stochastic processes around zero^15,17^. Wied^15^ has shown that under the null hypothesis, the distribution of the test statistic (after appropriate scaling of the difference in correlations) converges to a distribution based on the maximum sum of the absolute value of a set of standard independent Brownian bridges. However, if there is a correlation change, the sum of the observed differences will diverge strongly from zero, and the test statistic will exceed the cut-off of the null distribution.

For the toy example, the Cusum test generated the maximal test statistic at *T* = 105 (see Fig. 2e), implying a change point at *T* = 106 which is just 5 time points away from the first real change point at *T* = 101. However, similar to the Frobenius norm test, this test statistic falls in the middle of the null distribution (Fig. 2f) and is therefore declared as not significant. Inspecting the matrices used for the computation of the test statistic, which is the empirical correlation matrix up to *T* = 105, $${\hat{{\boldsymbol{\Sigma }}}}_{1:105}=[\begin{array}{ccc}1 & -0.04 & 0.17\\ -0.04 & 1 & 0.03\\ 0.17 & 0.03 & 1\end{array}]$$, and the overall empirical correlation matrix, $${\hat{{\boldsymbol{\Sigma }}}}_{1:250}=[\begin{array}{ccc}1 & 0.17 & 0.11\\ 0.17 & 1 & 0.08\\ 0.11 & 0.08 & 1\end{array}]$$, we see that the difference for the first two variables,

$${\hat{{\boldsymbol{\Sigma }}}}_{1:250}[1,2]-{\hat{{\boldsymbol{\Sigma }}}}_{1:105}[1,2]=0.17-(-0.04)=0.21,$$

is much too weak to represent the real abrupt correlation change of 0.7 that we have introduced. If the last 100 time points are discarded, converting the problem to a single change point case, Cusum would locate a significant change point at *T* = 98, however.

### KCP Permutation test

**Figure 3:**
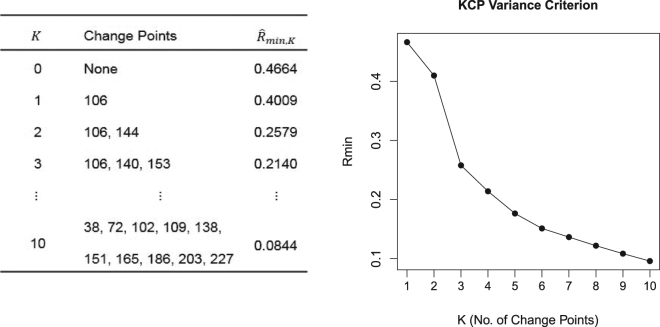
KCP change point solution and the ${\hat{R}}_{min}$-curve for the running correlations of the toy example. In (**a**), the change point locations and the variance criterion, ${\hat{R}}_{min,K}$ for every *K* (no. of change points) are tabulated. In (**b**), the ${\hat{R}}_{min}$-curve is exhibited, showing the expected downward trend as more change points are introduced in the data.

To solve the problems resulting from unwarranted pooling of different phases, we propose to use a permutation test based on the KCP (Kernel Change Point) detection method^11^. To focus on correlation changes, the raw data are first converted to running correlations by sliding a moving window across the time series and computing the correlation within each window. Then, KCP is applied to the running correlations to signal the most plausible change point locations when assuming 0 up to *K*_*max*_ change points. For deciding on the number of change points, a penalization scheme was proposed by Arlot *et al*.^11^. However, when this scheme is applied to the running correlations, the Type I error rate excessively exceeds the specified nominal rate. The penalized criterion, therefore, tends to choose *K* > 0 change points, even when no change points are present. We attribute this high Type I error rate to the strong serial dependence in the running correlations, which is a violation of the independence assumption implied by the penalized criterion. We therefore propose a different approach to determine whether there is at least one change point present in the running correlations. We look at the average within-phase variance of the running correlations, ${\hat{R}}_{min,K}$ [The formal definition of ${\hat{R}}_{min,K}$ is provided in Section 4.1.], which is yielded by KCP for each number of change points, *K*. This variance measure is obtained by searching for the change point locations. For a given *K*, KCP computes the average within phase variance for all possible change point locations and the optimal location(s) are the time points yielding the minimized variance measure. In Fig. 3a, we display the KCP solution for the toy example which is comprised of the optimal change point location(s) and the corresponding average within-phase variance, ${\hat{R}}_{min,K}$. KCP’s variance criterion drops as more change points are induced from the data, and this is exhibited by the downward trend of the ${\hat{R}}_{min}$-curve in Fig. 3b.

As generally holds for permutation tests^18,19^, the results for the original data are compared to those obtained when applying the same steps to several permuted versions of the data. According to the null hypothesis, no phases are present in the data, implying that we may permute it by randomly reordering the time points. The test then inspects two aspects which we describe further below: the overall variance of the running correlations, which equals ${\hat{R}}_{min,0}$ [${\hat{R}}_{min,0}$ is the within phase variance when there are no change points induced in the data (*K* = 0).] (variance test), and the drop in the average within-phase variance of the running correlations when change points are introduced (variance drop test). Since we conduct two tests, we use a Bonferroni correction to control the family wise error rate. Thus, the significance level of each test is set to $\frac{\alpha }{2}$.

#### Variance Test

**Figure 4:**
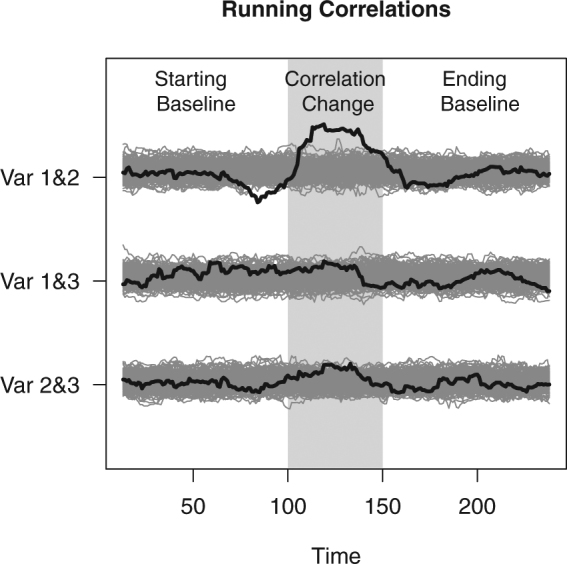
Running correlations of the toy example in comparison to the running correlations from permuted data. The black curves are the running correlations from the original data, while the gray curves are the running correlations from 1000 permuted data sets.

If the data contain a substantial correlation change, the overall variance of the running correlations for the original data will be larger than that of permuted data. This can be easily illustrated by looking at the running correlations of the toy example in comparison to those of permuted data in Fig. 4. The first two variables clearly exhibit more variation in their running correlations. Employing the variance test, we compare the overall variance of the running correlations, ${\hat{R}}_{min,K=0}$, to the distribution of the values ${\hat{R}}_{min,K=0,perm}$ (Fig. 2g). Evidently, the ${\hat{R}}_{min,K=0}$ for the toy example exceeds the 97.5^th^ percentile cut-off of the distribution obtained using the permuted data, therefore a significant correlation change can be declared.

The variance test becomes more sensitive when most running correlations change, generating a large ${\hat{R}}_{min,K=0}$. However, when only a few of the variables are correlating, sensitivity may decline. This is due to the fact that many non-changing running correlations will be included in the computation of ${\hat{R}}_{min,K=0}$. Consequently, the ${\hat{R}}_{min,K=0}$-value from the observed data will not be too different from ${\hat{R}}_{min,K=0,perm}.$

#### Variance Drop Test

With the variance drop test, we look at the maximal drop in ${\hat{R}}_{min,K}$ as a consequence of looking for an additional change point. We expect this drop to be dramatic if there is at least one underlying change point. For the permuted data, we do not expect a drastic improvement in fit if additional change points are extracted. We can thus perform a “variance drop” test which looks at the drop in the variance criterion, ${\hat{R}}_{min,K}-{\hat{R}}_{min,K-1}$, due to the introduction of an additional change point. We consider all drops for all *K* > 0, and retain the maximum one. For the toy example, this maximum drop in variance occurs at *K* = 2 (Fig. 3b). The permuted data is also scanned for the maximum drop in ${\hat{R}}_{min,K,perm}$. The variance drop test proceeds by comparing the maximum variance drop obtained from the original data to the distribution of the maximum variance drop in the permuted data. Considering the toy example once again, the maximum variance drop for the original data is clearly larger than the 97.5^th^ percentile of the corresponding distribution for the permuted data (Fig. 2h). Hence, using the variance drop test, we declare that there is a significant correlation change in the data. We note that it is possible that the maximum variance drop occurs at a *K* lower than the true number of change points in cases where some changes are more drastic than others. The variance drop test, therefore, cannot, on its own, determine the true number of change points, but only reveals that the time series contains at least one correlation change.

#### Combining both tests

Finally, the last step of the KCP permutation test is to look at the results of both the variance and the variance drop tests. Since there can be settings where one test can be more sensitive than the other, we combine their strengths by declaring significance whenever at least one of them is significant. The Type I error rate, however, is controlled using a Bonferroni correction as described above. For the toy example, both tests yield a significant result, hence the KCP permutation test successfully revealed the presence of the correlation change.

### Simulation studies

We set up two simulation studies to evaluate and compare the power of the four non-parametric tests under consideration–the Frobenius norm test, the Maximum norm test, the Cusum test, and the KCP permutation test–in detecting the presence of at least one correlation change. The first three tests are based on a single change point, while the KCP permutation test also considers scenarios with multiple change points. It is therefore interesting to investigate which test performs best in which settings [We note that studies to assess the performance of the mentioned tests were already done in the past. However, they were limited to the evaluation of absolute performance (e.g. Cusum tests’s performance across several settings was evaluated by Wied^15^) or compared to tests which are not specific for correlation change (e.g. Frobenius norm test’s performance was assessed by Barnett and Onnella^8^ in comparison to QuadForm and Likelihood-ratio, which are also sensitive to variance changes).].

In the first simulation study, we consider single and multiple change point cases, where there are baseline and event phases. The multiple change point settings include a return to the baseline phase after the event phase has finished (to be expected to occur in dynamic systems returning to their stable state after a perturbation^20^). Of course, there can also be cases when the dynamic system does not return to the baseline after an event and enters an entirely different phase instead. We examined settings such as these in the second simulation study.

### Simulation Study 1: Single and Multiple Change Points

For the first simulation study, we expect that in case of a single change point, the KCP permutation test will perform at least as well as single change point based tests. In the multiple change point setting, on the other hand, we hypothesize the KCP permutation test to have better performance based on the considerations we illustrated in the toy example.

#### Design and procedure

The simulated data were drawn from a multivariate normal distribution with means equal to zero and variances equal to 1. The time series is simulated to have a baseline (phase where variables are uncorrelated) and an event (phase where variables correlate). Of course, for settings with multiple change points, multiple baselines and events were patched together. The baseline always comprises *n* = 100 time points, but we varied the event sizes so that in some settings, they are shorter than the baseline. We also introduced noise (non-correlating) variables to mimic more difficult but realistic settings. The following factors were varied with 100 replicates per cell:

1. *Number of change points K*: 1, 2, 4
2. *Number of variables V*: 2, 3, 5, 7
3. *Number of correlating variables S*: ranges from 2 until *V* − 1 (the number of noise variables is *V-S*)
4. *Strength of correlation change* Δ*ρ*: 0.3, 0.5, 0.7 and 0.9
5. *Event phase size P*: 25, 50, 100

For computing the running correlations in the KCP permutation test, we used a moving window of 25 time points [We have implemented the KCP permutation test using varying window sizes: 25, 50, 75 and 100 time points, and Type 1 error was controlled at the chosen nominal rate for all window sizes (see Supplementary Fig. S4). Power, however, was maximal for the smallest window size, *w* = 25 (see Supplementary Fig. S5). We therefore recommend to use a small window size.] that was slid forward, one time point at a time, and 1,000 permutations were done for each data set. For Cusum, 10,000 realizations of the simulated Brownian paths were generated to approximate the null distribution as implemented in Wied^9^. For the Frobenius and Maximum norm analyses, 1,000 bootstrap samples were employed. The performance of the methods was assessed by looking at their power, which was computed as the proportion of data sets declared by the test as having at least one significant correlation change point. We set the Type I error rate for all tests at *α* = 0.05.

#### Results

**Figure 5:**
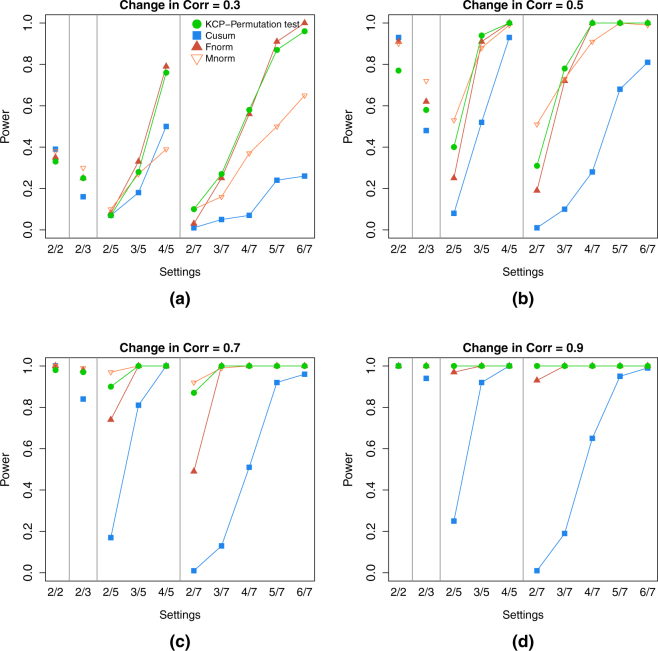
Power for settings with 1 correlation change point (event size = baseline size). The four panels, (a),(b),(c) and (d), correspond to the level of correlation change in the simulated data: 0.3, 0.5, 0.7 and 0.9, respectively. In every panel, the power of the KCP permutation test (in green points), Cusum (blue squares), and the Frobenius norm (dark red filled upward triangles) and Maximum norm tests (light red empty downward triangles), were plotted across the simulation settings on the x-axis, where each setting is written as (no. of correlating variables *S*)/(total no. of variables *V*).

For the single change point case, the KCP permutation test and the Frobenius norm test are the most powerful tests in almost all settings except in those with 2 correlating variables (Fig. 5). In these extremely noisy settings, the Maximum norm test proved to be the most sensitive. Cusum, on the other hand, was generally the least powerful test, except in settings with 2 variables, where it consistently performed best (or one of the best).

**Figure 6:**
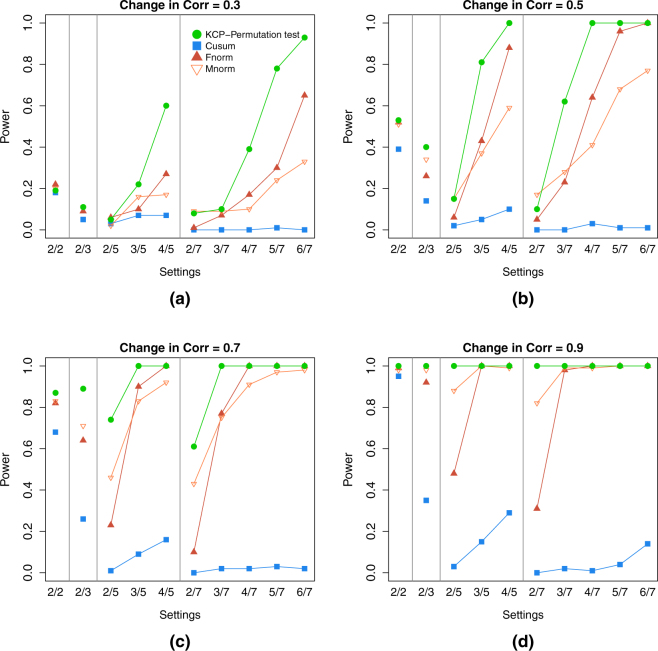
Power for settings with 2 correlation change points (event size = baseline size). The four panels, (a),(b),(c) and (d), correspond to the level of correlation change in the simulated data: 0.3, 0.5, 0.7 and 0.9, respectively. In every panel, the power of the KCP permutation test (in green points), Cusum (blue squares), and the Frobenius norm (dark red filled upward triangles) and Maximum norm tests (light red empty downward triangles), were plotted across the simulation settings on the x-axis, where each setting is written as (no. of correlating variables *S*)/(total no. of variables *V*).

For the multiple change points settings, Fig. 6, showing the two change point settings, clearly reveals that the KCP permutation test outperforms the other tests. It is also evident that the power of the other three tests drastically dropped in comparison to the 1 change point case. This is especially clear for the Cusum test, while the Frobenius and Maximum norm tests mostly deteriorated in settings with many noise variables.

**Figure 7:**
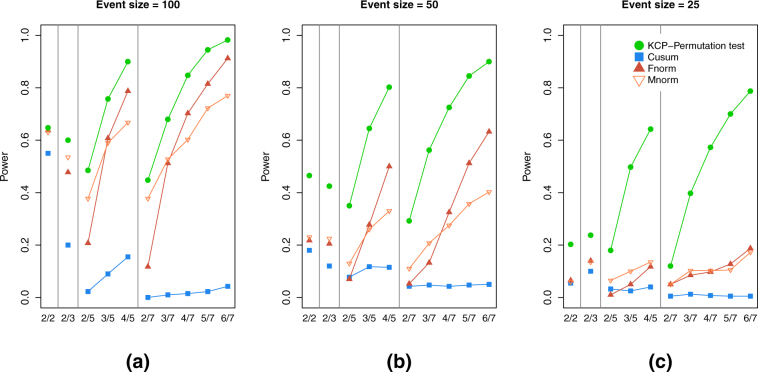
Power for settings with 2 correlation change points and varying event sizes: 100 (**a**), 50 (**b**) and 25 (**c**) time points. Power is averaged over all Δ*ρ* = 0.3, 0. 5, 0.7 and 0.9 In every panel, the power of the KCP permutation test (in green points), Cusum (blue squares), and the Frobenius norm (dark red filled upward triangles) and Maximum norm tests (light red empty downward triangles), were plotted across the simulation settings on the x-axis, where each setting is written as (no. of correlating variables *S*)/(total no. of variables *V*).

Figure 7 shows the effect of event size for the two change points case, revealing that for event phases with 50 and 25 time points only, the KCP permutation test still exhibited reliable power (≥0.80) in some settings. The Cusum, Frobenius norm and Maximum norm tests, on the other hand, have inadequate power in all settings. Similar results were obtained for settings with four change points (see Supplementary Figs S1-S2). Finally, we also examined how the tests performed in settings with no correlation change, and results revealed that all tests have fairly acceptable false positive rate around 0.05, which was the nominal rate implemented in the simulations (see Supplementary Fig. S3).

### Simulation Study 2: Multiple Change Points without Recurring Baseline Phases

So far, we have shown that by construction, the single change point tests pool several phases in case more than one change point is present in the data and that this might lead to underestimation of the real magnitude of a correlation change for time series with recurring baseline phases. However, in this second simulation study, we will look at scenarios where the time series do not return to a baseline. The first and the last phases are simulated to be dramatically different, such that despite the pooling of several phases, large correlation differences are still obtained. The goal is to determine whether single change point tests catch up with KCP permutation test in terms of power when implemented in time series where the correlation differences are not severely underestimated despite the pooling of phases.

#### Design and procedure

We simulated time series with three distinct phases. In the first phase, the variables are negatively correlated (ρ = −0.5). In the second phase, they become uncorrelated (ρ = 0), and in the third phase, they become positively correlated (ρ = 0.5). For the norm tests which look at the difference of correlation matrices before and after the change point, we expect that these settings are ideal. The reason is that the first and third phases are now very distinct rather than identical as was the case in the first simulation. This means that pooling the second and third phase generates even larger differences than merely comparing phase 1 and phase 2. For Cusum, the successive correlations which are first obtained at the beginning of the time series will be compared to the overall correlations, which are expected to be around 0 due to the pooling of the three phases. The test can potentially obtain sizeable correlation differences in these settings and gain in power, since the first successive correlations for the correlating variables will be around −0.5 (first phase has ρ = −0.5), in contrast to the first simulation study, where the first successive correlations for the correlating variables are around 0.

We note that we focused on the simplest multiple change point setting (i.e. the two change points setting) with the mentioned order of the distinct phases, since there are too many possibilities that one can configure when considering time series with non-recurring baseline phases. At the end, the goal is to simply examine how power changes when the pooling of phases is not problematic for the single change point tests. If their power is acceptable for this simple case, then it will hold in more than two change points settings with similar conditions. As with the first simulation study, the data was simulated from a multivariate normal distribution with zero means and unit variance. We remark that following the configuration of the time series we aim to simulate, we are constrained in introducing up to three negatively correlated variables only. Anything more than that will result to a non-positive definite covariance matrix. Hence, the number of correlating variables were limited to 3 variables only in case there are 5 or 7 variables in the time series. For this simulation study, therefore, the following factors were varied with 100 replicates per cell:

1. *Number of change points K*: 2
2. *Number of variables V*: 2, 3, 5, 7
3. *Number of correlating variables S*: 2, 3 (for *V* equals 5 and 7)

#### Results

**Figure 8:**
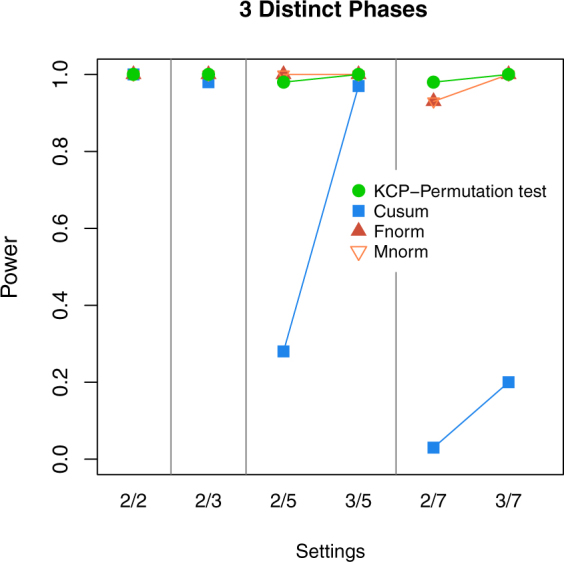
Power for simulation settings with 3 distinct phases. The power of the KCP permutation test (in green points), Cusum (blue squares), and the Frobenius norm (dark red filled upward triangles) and Maximum norm tests (light red empty downward triangles), were plotted across the simulation settings on the x-axis, where each setting is written as (no. of correlating variables *S*)/(total no. of variables *V*).

Results show that indeed, in settings with three distinct phases, the norm tests perform equally well as the KCP permutation test in terms of power (Fig. 8). Hence, we are able to confirm that in settings where pooling of phases does not lead to extreme underestimation of the test statistic, the norm tests catch up with the KCP permutation test in terms of power. Cusum’s power, on the other hand, was good when there are only 2 or 3 variables included and also when majority of the variables are correlating (3 out of 5). We remark that this was not observed for Cusum in the multiple change point settings of the first simulation study. We therefore see some improvement in its power, as expected, in case of three distinct cases. However, this is not as pronounced compared to the norm tests. Lastly, when there are many noise variables, Cusum proved to be the least sensitive test.

### Illustrative Application

#### Correlation change in EEG data

**Figure 9:**
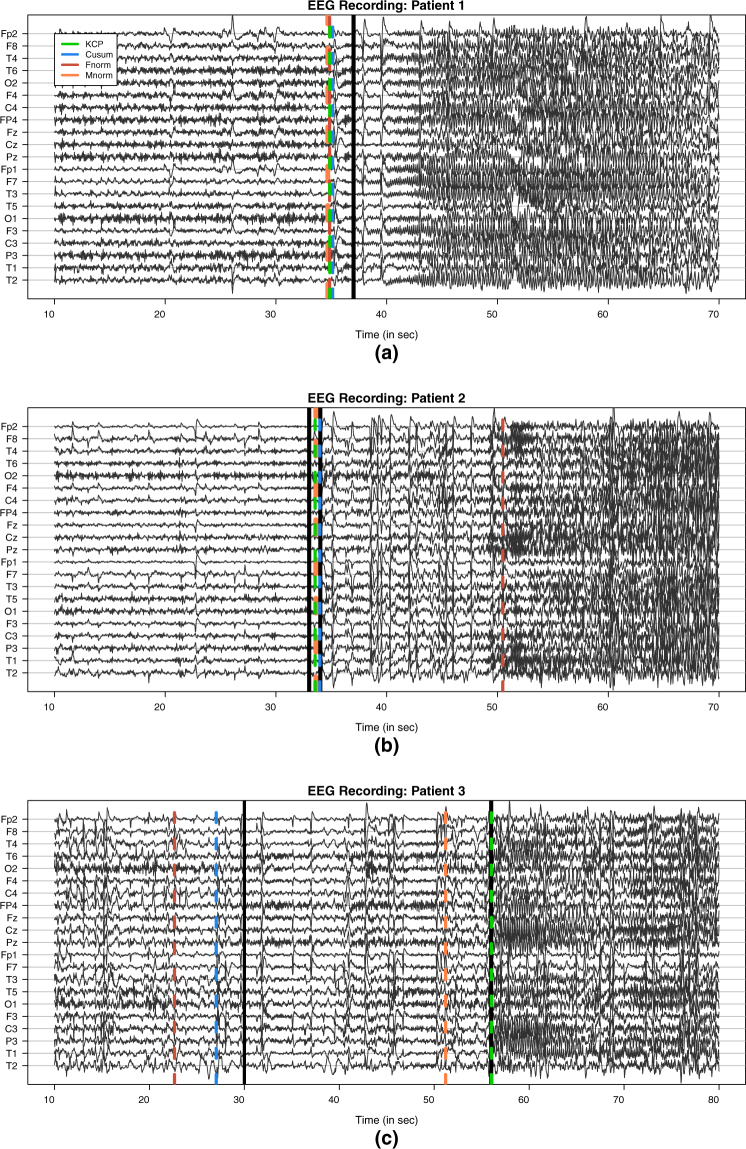
Recordings for 21 EEG channels in the vicinity of an epileptic seizure. The three panels correspond to recordings of three different patients, wherein the clinical and the electrophysiological (or EEG) onset are marked. The first black vertical line indicates the clinical onset, while the second one indicates the EEG onset. In (**a**), the methods signaled a common correlation change point around 2 seconds before the EEG onset (also the clinical onset for this patient) at *T* = 37 s. In (**b**), KCP, Cusum and Maximum norm yielded change points that coincide with the EEG onset at *T* = 34 s, while Frobenius norm obtained a much later change point at *T* = 50.5 s. This seemingly later detection, though, is reasonable since for this patient at *T* = 49 s, the ictal EEG pattern changes, exhibiting a rhythmic, high amplitude pattern, which is most pronounced at the Fp2, F8 and T2 channels. In (**c**), only KCP yielded a change point at the EEG onset (*T* = 56 s), while Maximum norm had a detection 5 seconds earlier. Frobenius norm and Cusum, on the other hand, have change points at *T* = 23 s and *T* = 27 s, respectively, which are closer to the clinical onset at *T* = 30 s.

The first illustrative application concerns epileptic seizure detection. To identify seizure onset, experts usually analyze the multi-channel scalp electroencephalogram (EEG) recordings, which often exhibit abnormal, hypersynchronous signals during seizure attacks^21^. For this purpose, several synchronization measures have been proposed and used^1,22^, however for this example, we focus on looking at changes in the linear correlations. We apply the KCP permutation test and the Frobenius norm, Maximum norm and Cusum tests to EEG recordings of three randomly chosen patients included in the study by Vergult *et al*.^23^. The anonymized dataset consisted of 70–90 s long multichannel data segments and a full text-based clinical report for each patient. The report included both the clinical and the electrophysiological onset time of the seizure, i.e. the time points when the first symptoms and the first EEG changes were observed, respectively, as well as the description of the ictal EEG (i.e., seizure pattern). Video EEG’s were obtained using a 21-channel recorder with a sampling frequency of 250 Hz. For our analysis, we downsampled the data at a frequency of 25 Hz, resulting in 1750 time points for the first and second patients who have 70 seconds of EEG recordings, and 2250 time points for the third patient who had a longer recording of 90 seconds (see Fig. 9). Each recording included the 30–40 seconds before the clinical onset, which is indicated by the first black vertical line in Fig. 9. The electrophysiological (or EEG) onset, on the other hand, is indicated by the second vertical line. For the first patient, the clinical onset and the EEG onset occur simultaneously, while for the second patient, these events are just one second apart. For the third patient, the onsets were 26 seconds apart however.

**Figure 10:**
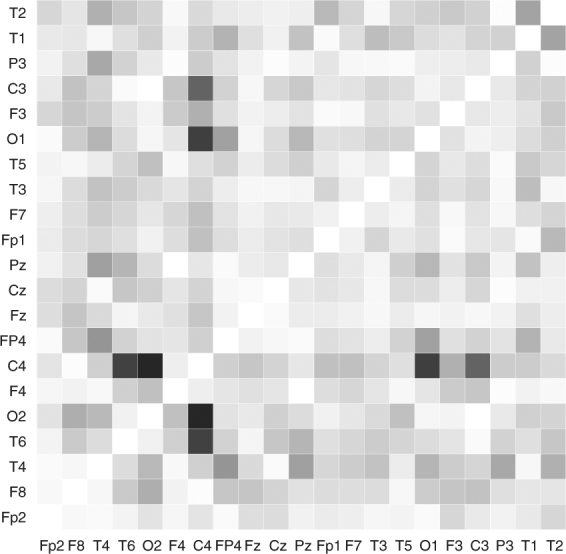
Heat map for the absolute correlation differences between the phases before and after the common change point yielded by the four compared methods for Patient 1. This common change point is 2 seconds before the EEG onset (which is also the clinical onset for this patient). Zero correlation change is indicated by a white cell and the intensity of the shading indicates the strength of the correlation change.

For the 3 EEG recordings, all four correlation change tests are significant (all p-values < 0.001). We proceed by examining the first detections. For the first patient, all methods indicate that a change point is present at *T* = 35 s, which is just 2 seconds before the EEG onset at *T* = 37 s (Fig. 9a). For the second patient, three of the four methods (the Maximum norm test, the Cusum test and the KCP permutation test) successfully detected a correlation change point during the EEG onset at *T* = 34 s (Fig. 9b). The Frobenius norm test, on the other hand, signals a change at *T* = 50.5 s, which is also relevant since, according to the clinical report, at *T* = 49 s, the ictal EEG pattern changes, exhibiting a rhythmic, high-amplitude pattern around 7 Hz, which is most pronounced at the Fp2, F8 and T2 channels. For the third patient, results diverge at first glance, as the obtained change points are far apart (Fig. 9c). However, for this specific example, the clinical onset is 26 seconds earlier than the EEG onset. The Frobenius norm test (*T* = 23 s) and Cusum test (*T* = 27 s) yielded change points proximal to the clinical onset at *T* = 30 s, while the Maximum norm test (*T* = 51 s) and the KCP permutation test (*T* = 56 s) signaled the EEG onset at *T* = 56 s. From Fig. 9, we conclude that correlation change points are proximal to the onset (either clinical or EEG onset), implying that the EEG channels can indeed exhibit significant shifts in correlation characterizing the onset of an epileptic seizure. For the first patient where one change point can be expected because the clinical onset and the EEG onset coincide, we further scrutinized the correlation difference between the before and after phases. Figure 10 displays the heat map for the absolute correlation differences (shading intensity indicates the strength of the correlation change), which is the same for all methods since the resulting change points coincide for this specific patient. The most sizable correlation shifts are observed for pairs involving the C4 channel (C4 and T6: 0.73, C4 and O2: 0.77, C4 and O1: 0.73, C4 and F3: 0.50 and C4 and C3: 0.66). We did not build heat maps for the before and after correlation difference for the next two patients since there can be multiple change points in the time series due to the different locations of the EEG and clinical onsets. This possibility is further supported by the change point locations differing across the considered methods. A better approach would be to locate the additional change points and compute the correlation differences for all the obtained phases. We stress, however, that locating multiple change points is beyond the scope of this paper.

In summary, we gain two important learnings from these EEG illustrative examples. First, correlation change detection methods can signal epileptic seizures as evidenced by the significance of the change points and their close proximity to either the clinical or EEG onset. Second, as seen for the second and third patients, different methods can yield different change point locations, and this can be an indication of the presence of more than one change point in the data. We note that artifact removal was not dealt with since our main goal is to simply exhibit that the methods pick up the presence of at least one correlation change in the time series regardless of the source. We only traced back the first detected change point for illustration’s sake and describe their location relative to the seizure onset or a relevant change in the EEG pattern. However, the reader should be well aware that the location of these first detections can be influenced by artifacts (e.g., muscle and eye artifacts). Thus, if the goal of the change point analysis is better localization of the seizure or earlier detection of onset, then appropriate data pre-processing should be done^23–25^.

#### Correlation change in depression data

The second empirical example examines the dynamical characteristics of depression-relevant momentary states that may act as early warning signals or indicators of a transition in or out of depressive episodes^26,27^. Based on complex dynamical system principles, previous research has shown that increased autocorrelation, variance and correlations between depression-relevant states or symptoms may reflect a tipping point that signal a change from healthy to depressed status or vice versa^20^. With the present analysis, we aim to test whether data from one patient contain such a correlation change point, and if so, whether it occurs before a known critical transition point in depressive status.

**Figure 11:**
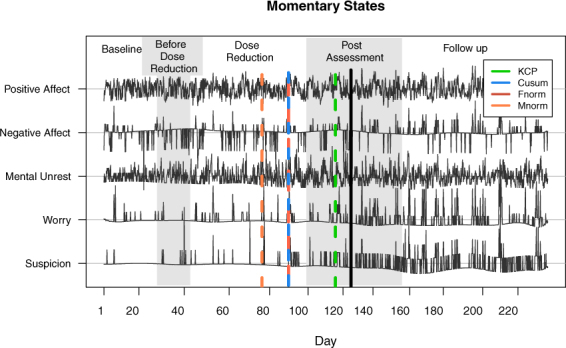
Momentary state ratings during a 239-day anti-depressant reduction experiment. The experimental phases are indicated by the varying background shading. The detected correlation change points (indicated by the dashed lines) provide some evidence supporting the critical slowing down phenomenon before a sudden critical transition, which was the depression relapse observed on Day 127 (indicated by the black vertical line).

The data are ratings of depression-relevant momentary states and other life experiences from a male participant diagnosed with major depressive disorder. The ratings were obtained up to ten times a day, while he was undergoing a 239-day anti-depressant reduction experiment^26^. There are five experimental phases: baseline (four weeks), before dose reduction (between zero and six weeks), dose reduction (eight weeks), post assessment (eight weeks) and follow up (twelve weeks). In the second and third phases, wherein dose reduction and eventual dose discontinuation were implemented, only the pharmacist was acquainted with the dose reduction scheme, while the participant and the researchers were blinded. On Day 127 of this experiment, a sudden transition was detected^26^, indicated by a black vertical line in Fig. 11, indicative of a relapse into depression. The original data set is publicly available and described in Kossakowski, Groot, Haslbeck, Borsboom and Wichers^28^.

Following Wichers and Groot^26^, we will focus on the five affect components: positive affect, negative affect, mental unrest, suspicion and worry (see Fig. 11). We implemented the methods discussed in this paper to find out whether there are indeed correlation change points preceding the critical transition point at Day 127. If this is the case, this contributes some evidence that critical slowing down occurs in depression.

**Figure 12:**
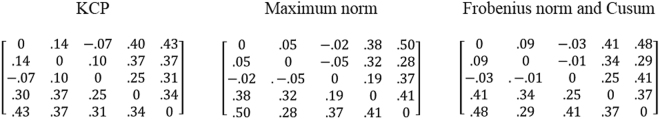
Correlation differences between the phases before and after the change point for the depression data. The difference matrices for the compared methods were obtained by subtracting the before-change-point correlation matrix from the after-change-point correlation matrix. Since the correlations before the change point are either positive or close to zero, the large positive differences imply that most of the correlations after the change point strengthened dramatically.

In Fig. 11, the change points that are detected by the four methods under study are displayed. Maximum norm test yielded the earliest detection at Day 76, then Frobenius norm and Cusum tests have a common detection at Day 89, and KCP permutation test had the latest detection at Day 116. In Fig. 12, we show the difference matrix obtained by subtracting the correlation matrix before the change point from the correlation matrix after the change point. Considering that all five variables are either uncorrelated or positively correlated before the change point, the common trend is that the correlations became stronger after the change point. The largest correlation changes occur for Worry and Suspicion (see the fourth and fifth columns, respectively).

In summary, our results agree with the previous findings of Wichers and Groot^26^, where more and stronger network linkages between the five momentary states were observed as the depression level escalated. Finally, we emphasize that the significance of these correlation change points, as well as their location, which precedes Day 127, the day of sudden transition in depression, provides evidence for depression exhibiting critical slowing down. We note, however, that correlations kept on rising even after the tipping point, which is somehow inconsistent with the critical slowing down theory, which states that correlation should decrease after the transition.

#### Correlation change in stocks data

**Figure 13:**
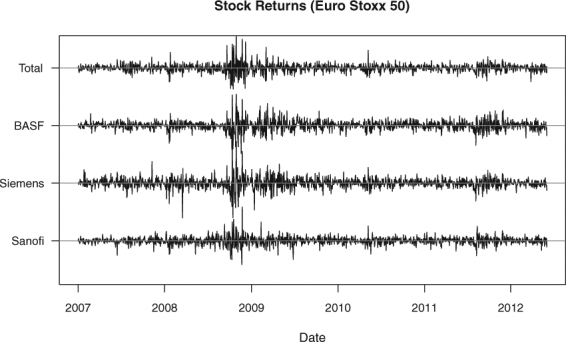
The time series data for the four stocks from Eurostoxx 50: BASF, Sanofi, Siemens and Total from 1 January, 2007 to 1 June, 2012.

Our final empirical application is on stock market analysis. In stock trading, financial managers aim to build a diversified portfolio containing unrelated stocks, so that a drop in one of them would not imply the same for the others. However, during a crisis, financial analysts refer to a phenomenon called “diversification meltdown”- where stocks would exhibit strong correlations as they drop together in value. Galeano and Wied^17^ have found some evidence for this phenomenon using the Cusum method to the returns data [A return is defined as follows: (today’s price - yesterday’s price)/(yesterday’s price).] of four stocks in the Eurostoxx 50 (Total, BASF, Siemens and Sanofi) from January 1, 2007 to June 1, 2012. We obtained a similar data set from the Datastream database^29^ (Fig. 13), to which we applied the four compared methods. Our goal is to detect whether there is indeed a significant correlation change occurring in the data and to assess whether these occur close to a known financial crisis.

For this data, all methods confirmed that a significant correlation change is present. The most important change points [In Galeano and Wied^17^, the most important change point was located at *T* = 443, which is 7 time points later than the estimate we generated after implementing the same method. We still consider these results similar, though, because of the closeness of these estimates and of the Cusum test statistics (ours:6.4519, Galeano and Wied^17^: 6.3280). We note that we obtained the data directly from the Datastream database, and this data might have minimal differences with the one analyzed by Galeano and Wied^17^. Another source of the differences in the generated test statistic value is the bootstrapping procedure done to estimate the scaling matrix, ${\hat{{\boldsymbol{E}}}}^{-\frac{1}{2}}$ (see Methods section for details).] from the KCP permutation test and the Cusum test are very close at *T* = 436 and *T* = 437, respectively. Henceforth, we will consider these change points as a common detection at *T* = 436 (September 2, 2008), so results below can be presented concisely. The Maximum norm test had an earlier detection at *T* = 275, while the Frobenius norm test pointed at an even earlier change point at *T* = 135. It is interesting to note that the change point that results from the Frobenius norm test is identical to the second most important change point obtained by Galeano and Wied^17^ when they implemented the Cusum method in a sequential way to search for multiple change points in the present data set.

**Figure 14:**
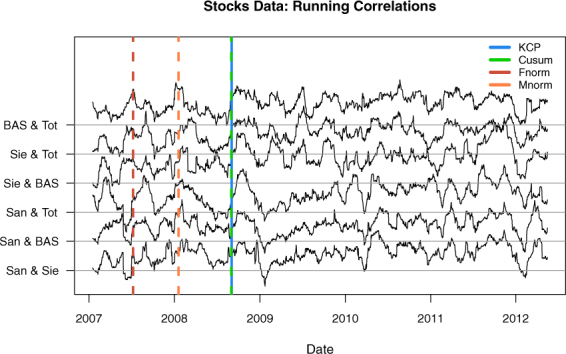
Running correlations (window size = 25 days) for the stocks data and the most important change points obtained by the KCP, Cusum and Frobenius and Maximum norm tests. Both the KCP and Cusum tests yield a change point at *T* = 436, where correlations drastically increased for almost all pairs. The Frobenius and Maximum norm tests, on the other hand, revealed ealier change points at *T* = 135 and *T* = 275, respectively.

Since there are only a few variables in this data, we are able to examine the running correlations in detail (Fig. 14). A remarkable rise in correlations is indeed observed at the change point, *T* = 436, for almost all pairs. At *T* = 275, high peaks were also observed, and at *T* = 135, pairs associated with Sanofi and Siemens exhibited the most pronounced changes. All in all, our results confirmed the findings of Wied and Galeano^19^ regarding the occurrence of increased correlations between stocks during a crisis. The most important change point for KCP and Cusum (*T* = 436: September 2, 2008), for instance, is roughly two weeks before the collapse of Lehman Brothers on September 15, 2008. Also around this period, other important financial institutions collapsed such as Merrill Lynch, and bank bailouts for Bear Stearns, Bank of America and Bankia took place^17^.

We conducted two additional analyses to examine how robust the results generated by the different methods are. First, we included five randomly selected stocks from the Eurostoxx 50 to an extended version of the time series (including time points until December 31, 2016). The new data sets, thus, contained 9 variables (4 original stocks and 5 randomly selected stocks) with 2,609 time points. This process was repeated 100 times, leading to 100 new data sets. Depending on which stocks are added, different scenarios are possible. First, the added stocks can have the same change points as the original four stocks, magnifying the correlation changes. Second, they can have their own change points at a different location, implying more change points for the whole time series. Third, they can contain no change points, implying that they are just noise variables after all. These last two conditions would be more difficult settings, in terms of power, as previously shown in the simulation study. Out of the 100 data sets, the KCP permutation test indicated a significant correlation change 91 times. The other methods yielded lower yet acceptable rates as well (Frobenius norm: 83%, Maximum norm test: 82%, Cusum test: 80%).

The second extra analysis had almost the same configuration as the first one. However, instead of adding a set of stocks, we added five noise variables [The noise variables were simulated from a multivariate normal distribution with zero means and unit variance.] to see whether the methods will still pick up the same correlation change as in the original time series despite the noise variables outnumbering the original variables. For 100 simulated data sets drawn, we have obtained very extreme results: The Cusum test did not declare a significant correlation change in any of them, while the three other methods signaled a significant correlation change for all of them. The results therefore confirm the results from our simulation study, that is, the Cusum test can suffer from a loss of power once there are too many noise variables. The other three methods, on the other hand, seemed to be very robust against this.

## Discussion and Conclusion

Existing methods to signal the presence of correlation changes in multivariate time series compute their test statistics assuming that the time series contains only a single change point. Through a toy example, we have clearly established that these single change point based tests may suffer from low power in case pooling some of the phases during the optimization of the test statistic leads to underestimation of the true correlation change occurring in the data. We deem this constraint to be an important methodological limitation as it is reasonable to assume that many dynamic systems have a stable state, where they tend to go back to maintain equilibrium. For instance, we can consider the non-seizure period as the stable state in an epileptic seizure data. In stock analysis, periods before and after diversification meltdown can be viewed as the equilibrium states. To bridge this gap, we proposed the KCP permutation test, which is based on a non-parametric change point detection method capable of estimating several change point locations simultaneously. By looking at how the average within-phase variance improves after inducing additional change points and thus additional phases, the test signals whether at least one correlation change point is present.

The proposed KCP permutation test emerged as the most powerful test in two simulation studies. It was the most reliable test in multiple change point settings where the baseline phase recurs (settings where pooling some phases during the optimization of the test statistic may become problematic). When the situation becomes more difficult in that the event sizes are reduced, the KCP permutation test still exhibited acceptable power whereas the other tests did not. In settings where pooling some phases does not occur (single change point case) or is not so problematic (multiple change point case where baseline does not recur), the proposed test performed as good as the norm tests (Frobenius norm and Maximum norm). We can therefore generally recommend the use of the KCP permutation test.

We also emphasize its robustness against noise variables in comparison to other tests. We note, however, that in extremely noisy settings where there are only two correlating variables, the Maximum norm test can be more sensitive. This finding makes sense since this test was actually proposed to signal this type of correlation changes^8^. Putting the two norm tests side by side, the Frobenius norm test quickly picks up in sensitivity when there are at least three variables correlating and eventually becomes the more powerful test once the noise variables are outnumbered by the involved ones. Our results also revealed that Cusum was the least sensitive of all tests compared, yet we also note that the settings presented had small sample sizes, while Cusum was proposed for time series from the stock analysis domain where thousands of time points are easily available^15^.

Applying the methods to real data, highly meaningful results were obtained. First and foremost, the conclusions converged to some extent. For the epileptic seizure data, all tests indicated significant correlation changes indicative of the clinical and/or the EEG onset. For the depression data, they all detected significant increase in correlations before a critical transition point. However, the increase went on until the end of the time series. In summary, we have a mixed evidence that depression exhibits critical slowing down. For the stocks data, they also pinpointed significant correlation changes close to the occurrence of a well-known financial crisis. The location of the first change points, though, did not always coincide. In fact, for only one out of the five illustrative time series (3 EEG, 1 depression and 1 stocks) we examined, did all the methods pinpoint a common change point. For the rest of the data, there was a subset of methods pinpointing a certain location while one (or another subset of) method(s) detects a different one. The membership of these subsets is also not consistent. From these findings, one might conclude that different location estimates obtained from different methods can possibly signal the presence of multiple change points (as with the EEG data examples 2 and 3). This would mean that the first change point detected by some methods can possibly be revealed by other methods when they are used to detect multiple change points. One should realize, however, that it remains hard to judge whether a change point at a different location is indeed a signal for an additional change point or simply a biased estimate.

This brings us to the fact that this paper is just a first step in comparing the different methods in detecting correlation change points. We only compared their power in detecting the presence of at least one correlation change point since this is the first crucial step for all methods to decide whether or not to proceed with the entire change point detection process. Yet the task of detecting all correlation change points in the time series still requires two additional but intertwined steps which can be further studied: estimating the number and the location of the change points. Towards this end, the norm methods can be implemented sequentially until no significant change point is found. However, since in each sequential step the significance of the change point should be tested, proper correction of the Type 1 error rate should be done to avoid too many false detections. Similarly, Cusum can be implemented sequentially to detect multiple change points. Galeano and Wied^17^ already proposed an algorithm for Cusum, wherein aside from correcting for the Type 1 error due to multiple testing, another refinement step is introduced to further prune the change points. We note, however, that Cusum was the least powerful in detecting if there exists at least one correlation change point in our simulation studies, which means that it already has the disadvantage of not being able to detect any change point at all in difficult settings in comparison with the norm methods and KCP. Since KCP will, for any *K*-value provided by the user, optimize the location of all *K* change points simultaneously, it is necessary to develop a rule which can optimally estimate *K*. This would allow a direct comparison of KCP to Cusum and the norm methods in terms of recovering the number and location of true change points. Once this direct comparison is possible, one can examine which method yields the least false detections and the least biased change point estimates. Furthermore, possible methodological drawbacks which can be improved to yield more reliable change point solutions, can be identified. From a more applied perspective, one can also investigate how accurate the location estimates are when the methods are used in more complex yet still realistic settings, for instance, when the correlation change across variables is not simultaneous or when it occurs gradually (in contrast with the abrupt change assumption in change point detection methods).

All in all, we have shown that the KCP permutation test on the running correlations exhibits a reasonable performance in comparison to recently proposed correlation change tests. One of the major advantages of our approach is that we may replace the running correlation coefficient with any other measure of dependence. For example, in EEG analysis, the PLV (Phase Locking Value)^30^ and the PLI (Phase Lag Index)^31^ pick up non-linear dependencies (to which simple linear correlation measures such as the Pearson’s correlation are blind). Obviously, it should then be investigated in future work whether the performance of the permutation based test extend to these new measures. In fact, since KCP can be applied to the raw data or any other derived time series (as we have shown for running correlations), the permutation based test can potentially work well to test for changes in mean, variance and possibly other parameters.

In conclusion, we have proposed the KCP permutation test for signaling the presence of a correlation change in a multivariate time series. This test proved to be more sensitive in settings where the single change point based test may break down: multiple change points scenario with recurring baseline phases and settings with more change points and/or more noise variables. We therefore generally recommend its use.

## Methods

### The KCP (Kernel Change Point) permutation test

KCP segments a multivariate time series into homogeneous phases by pooling adjacent observations that are similar in a phase. Thus, for a multivariate time series ***X*** = {***X***_1_, ***X***_2_, …, ***X***_*n*_}, where ***X***_*i*_ denotes the variable scores at time point *i*, a measure of similarity for each ***X***_*i*_ and ***X***_*j*_ is computed using the following Gaussian kernel,

1 $$Gk({{\boldsymbol{X}}}_{i},{{\boldsymbol{X}}}_{j})=\exp (\frac{-\parallel {{\boldsymbol{X}}}_{i}-{{\boldsymbol{X}}}_{j}{\parallel }^{2}}{2{h}^{2}}).$$

This similarity measure yields a value close to 1 when the observations are very similar and approaches 0 when they are dissimilar. The denominator includes the bandwidth, *h*, which is computed by taking the median Euclidean distance for all ***X***_*i*_’s. Since in this paper, we implement the method to the running correlations, we employ the similarity measure,

2 $$Gk({{\boldsymbol{R}}}_{i},{{\boldsymbol{R}}}_{j})=\exp (\frac{-\parallel {{\boldsymbol{R}}}_{i}-{{\boldsymbol{R}}}_{j}{\parallel }^{2}}{2{h}_{R}^{2}}),\,$$

which is the similarity between the running correlations for time points *i* and *j*. As can be seen above, *Gk*(***R***_*i*_, ***R***_*j*_) is computed in exactly the same way as *Gk*(***X***_*i*_, ***X***_*j*_), however the vectors of raw data, ***X***_*i*_ and ***X***_*j*_, are replaced by the vectors of running correlations, ***R***_*i*_ and ***R***_*j*_, and the bandwidth, *h*_*R*_, is obtained by computing the median Euclidean distances between all ***R***_*i*_’s. The vector of running correlations, ***R***_*i*_, is derived by computing the pairwise correlations in a window, *w*_*i*_, which includes *w* subsequent observations, with time point *i* as the midpoint. Thus, if there are *V* variables, then ***R****i* can be written as $${{\boldsymbol{R}}}_{i}=[\begin{array}{c}{w}_{i,1}\\ {w}_{i,2}\\ \vdots \\ {w}_{i,\frac{V(V-1)}{2}}\end{array}]$$, where $\frac{V(V-1)}{2}$ is the total number of variable pairs possible.

Given the number of change points, *K*, KCP quantifies the variance in the running correlations by computing the average within-phase variance,

3 $$\hat{R}({\tau }_{1},\,{\tau }_{2},\,\ldots ,\,{\tau }_{K})=\frac{1}{n}\sum _{p=1}^{K+1}{\hat{V}}_{p},{\tau }_{1},{\tau }_{2},\ldots ,{\tau }_{K},$$

where

4 $${\hat{V}}_{p,{\tau }_{1},{\tau }_{2},\ldots ,{\tau }_{K}}=({\tau }_{p}-{\tau }_{p-1})-\frac{1}{{\tau }_{p}-{\tau }_{p-1}}\sum _{i={\tau }_{p-1}+1}^{{\tau }_{p}}\sum _{j={\tau }_{p-1}+1}^{{{\rm{\tau }}}_{p}}Gk({{\boldsymbol{R}}}_{i},{{\boldsymbol{R}}}_{j}),$$

is the within-phase scatter for phase, *p*, and *τ*_*p*_ denotes the last observation in phase *p*, implying a change point at *τ*_*p*_ + 1. The within-phase scatter takes on a small value when the running correlations within a phase are very similar as a consequence of the rightmost term becoming more negative with larger similarities. Since for a given number of change points, *K*, the goal is to obtain *K* + 1 phases within which the running correlations are as homogeneous as possible, KCP searches for

5 $${\hat{\tau }}_{1},{\hat{\tau }}_{2},\ldots ,{\hat{\tau }}_{K}=\arg \,min\,\hat{R}({\tau }_{1},{\tau }_{2},\ldots ,{\tau }_{K})=\arg \,min\,\frac{1}{n}\sum _{p=1}^{K+1}{\hat{V}}_{p},{\tau }_{1},{\tau }_{2},\ldots ,{\tau }_{K},$$

which determines the optimal change point locations, ${\hat{\tau }}_{1}$ + 1, ${\hat{\tau }}_{2}$ + 1,…, ${\hat{\tau }}_{K}$ + 1. The minimal $\hat{R}({\tau }_{1},{\tau }_{2},\ldots ,{\tau }_{K})$, which we denote as ${\hat{R}}_{min,K}$, is the minimized average within-phase variance used in the KCP permutation test. However, in the context of the test, we simply referred to it as the average within-phase variance since the minimization process is implied when running KCP. For a concrete example, we refer the reader to Fig. 3a in the Results section, where we tabulated the ${\hat{R}}_{min,K}$-value and the optimal change point locations for every *K* considered for the toy example.

### Variance Test

The first subtest of the KCP permutation test is the “variance test,” which examines the over-all variance of the running correlations, obtained by computing, ${\hat{R}}_{min,K=0}$, the average within-phase variance when no change points are induced. When there is a substantial correlation change in the data, more variations are expected in the original time series than in the permuted counterpart. The variance test therefore proceeds by comparing the original ${\hat{R}}_{min,K=0}$- values to the distribution of the values ${\hat{R}}_{min,K=0,perm}$ for *B* permuted data sets, with *B* being large (e.g., *B* = 1,000). The exact p-value for this test is given by,

6 $${p}_{variancetest}=\frac{{\rm{\#}}({\hat{R}}_{min,K=0,perm} > {\hat{R}}_{min,K=0})}{B},$$

the proportion of permuted data sets with the overall variance exceeding that of the original data.

### Variance drop test

The KCP solution, by construction, improves the ${\hat{R}}_{min,K}$-value as *K* is increased. When more change points are induced, the obtained phases become more homogeneous, leading to a smaller average within-phase variance. However, if there are no real change points underlying the data, this reduction in ${\hat{R}}_{min,K}$ would not be dramatic. Hence, to signal the presence of true change points, the variance drop test looks at the drop in the average within-phase variance as a consequence of adding another change point. It examines the drops, ${\hat{R}}_{min,K}-{\hat{R}}_{min,K-1}$, for all *K* > 0, and compares the maximum to the distribution of maximum drops obtained from the permuted data. Given *B* permutations, the *p*-value is obtained by,

7 $${p}_{variancedroptest}=\frac{{\rm{\#}}(max\,variance\,dro{p}_{perm} > \,max\,variance\,drop)}{B},$$

which is the proportion of permuted data sets for which the maximum variance drop exceeds that of the original data.

### Combination of both tests

Since there are two tests conducted, the significance level for each test is set to $\frac{\alpha }{2}$, employing a Bonferroni correction to control the overall false detection rate at α. Finally, the KCP permutation test declares a significant correlation change in the data whenever at least one of the variance or variance drop tests is significant, or more precisely, ${p}_{variancetest}\,\,$and/or ${p}_{variancedroptest}$ are/is less than $\frac{\alpha }{2}$.

### Single change point based methods

#### Frobenius and maximum norm

**Frobenius norm** The Frobenius norm test aims to find the change point, ${\hat{\tau }}_{1}+1$, such that the before-correlation matrix, ${{\boldsymbol{\Sigma }}}_{1:{\hat{\tau }}_{1}}$, is maximally different to the after-correlation matrix, ${{\boldsymbol{\Sigma }}}_{{\hat{\tau }}_{1+1}:n}$. The motivation is simple: If there exist a change point, then the observations before it will have a correlation structure which is different from that of the observations after it. Therefore, the squared Frobenius norm of the differences between the before and after correlation matrices, $d({\tau }_{1})=\parallel {{\boldsymbol{\Sigma }}}_{1:{\tau }_{1}}-{{\boldsymbol{\Sigma }}}_{{{\rm{\tau }}}_{1}+1:n}{\parallel }_{F}^{2}$, is computed for all *τ*_1_ ∈ {1, 2, 3, …, *n*}, and the most plausible change point ${\hat{\tau }}_{1}+1$ is determined by

8 $${\hat{\tau }}_{1}={\rm{\arg }}\,{\rm{\max }}\,{z}_{{\hat{\tau }}_{1}}={\rm{\arg }}\,{\rm{\max }}\,\frac{\,d({\tau }_{1})-\hat{\mu }({\tau }_{1})}{\sqrt{{\hat{\sigma }}^{2}({\tau }_{1})}},$$

where ${z}_{{\hat{\tau }}_{1}}\,$is a standardized *d*(*τ*_1_), and is the test statistic for this test. The terms used for the standardization is obtained from *B*(e.g., *B* = 1,000) bootstrap samples. If the time series is independent, a bootstrapped sample is equivalent to a random draw with replacement from all time points of ***X***. However, if there is some autocorrelated process in the data, the method can also employ the sieve bootstrap^32^. The term, $\hat{\mu }({\tau }_{1})=\frac{1}{B}\sum _{b=1}^{B}{d}^{b}({\tau }_{1})$, is therefore the mean of all squared Frobenius norms at ${\hat{\tau }}_{1}$, and ${\hat{\sigma }}^{2}({\tau }_{1})=\frac{1}{B-1}\sum _{b=1}^{B}{({d}^{b}({\tau }_{1})-\hat{\mu }({\tau }_{1}))}^{2}$ is the corresponding sample variance. The p-value for the test statistic is computed by comparing ${z}_{{\hat{\tau }}_{1}}$ to the distribution of the bootstrapped z-scores, ${z}_{{\hat{\tau }}_{1}^{b}}$,

9 $${p}_{Fnormtest}=\frac{{\rm{\#}}({z}_{{\hat{\tau }}_{1}^{b}} > {z}_{{\hat{\tau }}_{1}})}{B}.$$

These ${z}_{{\hat{\tau }}_{1}^{b}}$’s are obtained by computing the z-scores for all possible change point locations in each bootstrap and then retaining the maximal one. If the p-value is less than the significance level, or equivalently, when there are too few bootstrapped samples yielding a z-score greater than that of the original data, the Frobenius norm test declares ${\hat{\tau }}_{1}+1$ as a significant correlation change point.

Maximum norm: The Maximum norm test is performed in the same way. The only difference is that instead of looking at the squared Frobenius norm of the difference matrix, ${{\boldsymbol{\Sigma }}}_{1:{\tau }_{1}}-{{\boldsymbol{\Sigma }}}_{{{\rm{\tau }}}_{1}+1:n}$, the Maximum norm considers only the maximum element (in absolute value) from the same matrix. Hence, Maximum norm is expected to pick up the largest correlation changes occurring in the subset of monitored variables.

#### Cusum

While the Frobenius norm procedure maximizes the differences in the correlation matrices before and after a candidate change point, Cusum looks at the differences between the successively computed correlations and the overall correlations. The motivation for the Cusum test is that when there is no correlation change in the data then the difference between the current correlations and the overall correlations will fluctuate around zero but not excessively, as with standard Brownian bridges^15,33^. Thus, for a time series with *V* variables, implying $\frac{V(V-1)}{2}$ pairwise correlations, Cusum searches for the location, ${\hat{\tau }}_{1}$, wherein the vector of current correlations, $${{\boldsymbol{p}}}_{1:{\hat{\tau }}_{1}}=[\begin{array}{c}{p}_{1:{\hat{\tau }}_{1},1}\\ {p}_{1:{\hat{\tau }}_{1},2}\\ \vdots \\ {p}_{1:{\hat{\tau }}_{1},\frac{V(V-1)}{2}}\end{array}]$$, computed using only the observations, ${{\boldsymbol{X}}}_{1:{\hat{\tau }}_{1}}$, are as distant as possible to the vector of overall correlations, $${{\boldsymbol{p}}}_{1:{\rm{n}}}=[\begin{array}{c}{p}_{1:{\rm{n}},1}\\ {p}_{1:{\rm{n}},2}\\ \vdots \\ {p}_{1:{\rm{n}},\frac{V(V-1)}{2}}\end{array}]$$, which are computed using all data points.

The ${L}_{1}$-norm (||·||_1_) of the difference vector, ${{\boldsymbol{P}}}_{{\tau }_{1},n}$, is obtained by taking the sum of the absolute values of all its elements (i.e. the differences). This quantity is computed for all *τ*_1_ ∈ {2, 3, …, *n*}, and the most plausible change point, ${\hat{\tau }}_{1}+1$, is determined by

10 $${\hat{\tau }}_{1}={\rm{\arg }}\,{\rm{\max }}\,\parallel {{\boldsymbol{P}}}_{{\tau }_{1},n}{\parallel }_{1}={\rm{\arg }}\,{\rm{\max }}\,\parallel {{\boldsymbol{p}}}_{1:{\tau }_{1}}-{{\boldsymbol{p}}}_{1:{\rm{n}}}{\parallel }_{1}.$$

In line with the fluctuation test framework, the correlation change is determined to be significant whenever the difference between the successive and the overall correlation becomes too large or when the successive correlations fluctuate too much^15^. As derived by Wied^15^, the formal test statistic is given by

11 $${A}_{{\hat{\tau }}_{1}}=\frac{{\hat{\tau }}_{1}}{\sqrt{n}}\parallel {\hat{{\boldsymbol{E}}}}^{-\frac{1}{2}}{{\boldsymbol{P}}}_{{\tau }_{1},n}{\parallel }_{1},$$

where $\hat{{\boldsymbol{E}}}$ is the empirical covariance matrix of the vector of overall correlation estimated using a moving block bootstrap [We refer the reader to Wied^15^ and Galeano and Wied^17^ for the detailed description of this technique.]. This test statistic is compared to the distribution of

12 $$A=ma{x}_{0\le s\le 1}||{\boldsymbol{B}}{{\boldsymbol{r}}}^{\frac{V(V-1)}{2}}(s)|{|}_{1},$$

where ${\boldsymbol{B}}{{\boldsymbol{r}}}^{\frac{V(V-1)}{2}}(s)$ is a vector of $\frac{V(V-1)}{2}$ standard Brownian bridges [The values for s indicates the mapping to the independent standard Brownian bridge, which is defined at [0, 1]]. As suggested by the same author, the distribution of *A* can be approximated by simulating the paths of the Brownian bridge on fine grids. We refer the reader to Wied^15^ for the details of the derivation of the test statistic and the proofs for the distribution under the null. The p-value is given by

13 $${p}_{Cusumtest}=\frac{{\rm{\#}}(A > {A}_{{\hat{\tau }}_{1}})}{B},$$

wherein *B* is the number of samples generated to approximate the paths of the Brownian bridges. The change point, ${\hat{\tau }}_{1}+1$, is declared significant if ${p}_{Cusumtest}$ is less than the significance level set, or the test statistic is extremely large such that there are only a few samples from the null distribution exceeding it.

### Data availability

All codes used as well as the EEG and the stocks data are available upon request from the corresponding author. The depression data is publicly available at https://openpsychologydata.metajnl.com/articles/10.5334/jopd.29/.

## Electronic supplementary material

Supplementary Figures
