# Supplementary material for: Testing for the Presence of Correlation Changes in a Multivariate Time Series: A Permutation Based Approach
Source: Sci Rep. 2018 Jan 15;8:769. doi: 10.1038/s41598-017-19067-2 (PMC5768740; doi:10.1038/s41598-017-19067-2)
Supplement: Supplementary file 1 — Supplementary Figures [file 41598_2017_19067_MOESM1_ESM.pdf]

Testing for the Presence of Correlation Changes in a Multivariate Time Series: A Permutation Based Approach

Jedelyn Cabrieto[1*], Francis Tuerlinckx[1], Peter Kuppens[1], Borbála Hunyadi[2,3] and Eva Ceulemans[1]

[1]Research Group of Quantitative Psychology and Individual Differences,

KU Leuven – University of Leuven, Belgium

[2]Department of Electrical Engineering (ESAT), STADIUS Center for Dynamical Systems,
Signal Processing and Data Analytics,
KU Leuven – University of Leuven, Belgium

[3]imec, Leuven, Belgium

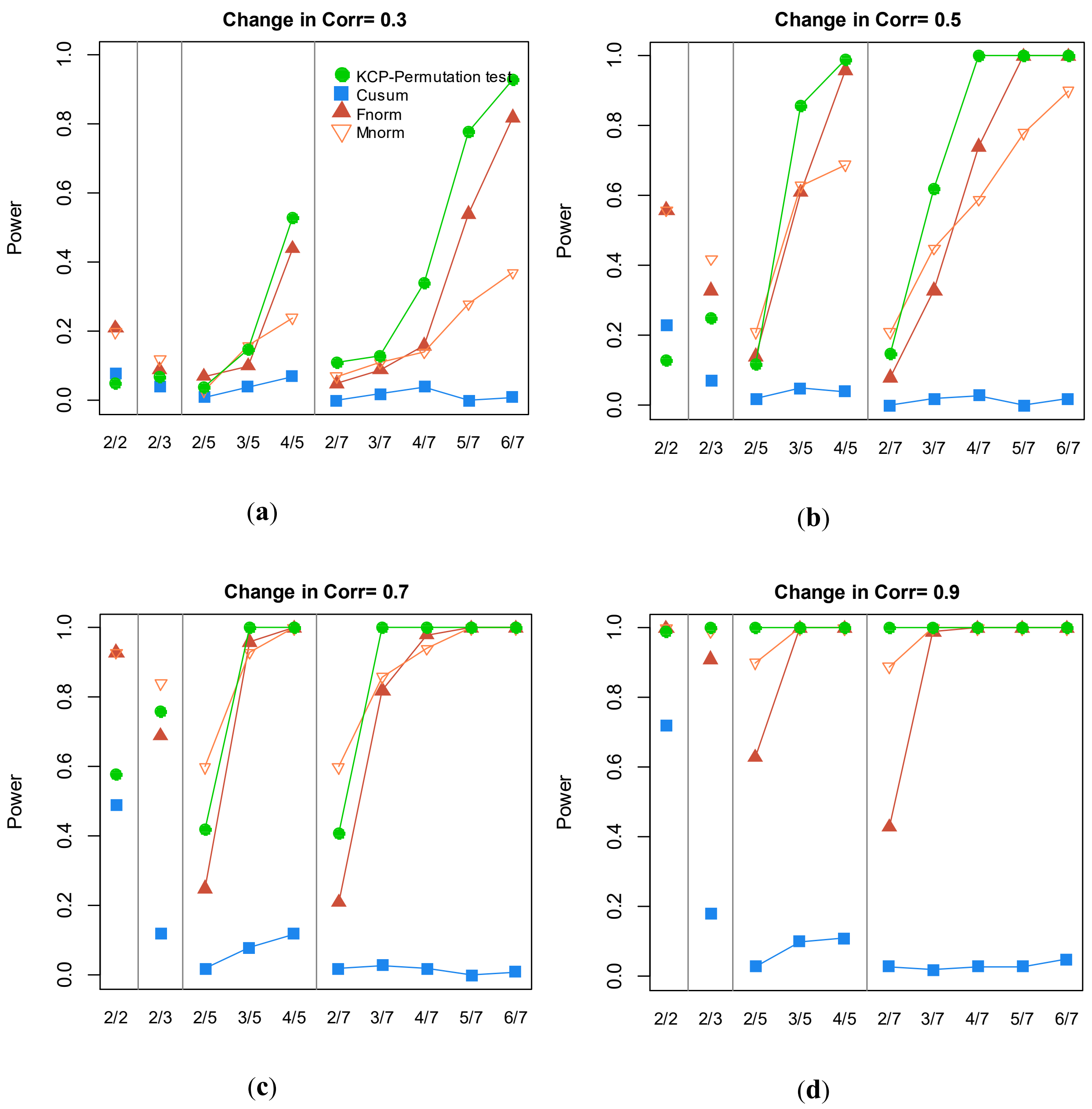

Figure S1. Power for settings with 4 correlation change points (event size = baseline size). The four panels, (**a**), (**b**), (**c**) and (**d**), correspond to the level of correlation change in the simulated data: 0.3, 0.5, 0.7 and 0.9, respectively. In every panel, the power of the KCP permutation test (in green points), Cusum (blue squares), and the Frobenius norm (dark red filled upward triangles) and Maximum norm tests (light red empty downward triangles), were plotted across the simulation settings on the x-axis, where each setting is written as (no. of correlating variables *S*)/(total no. of variables *V*).

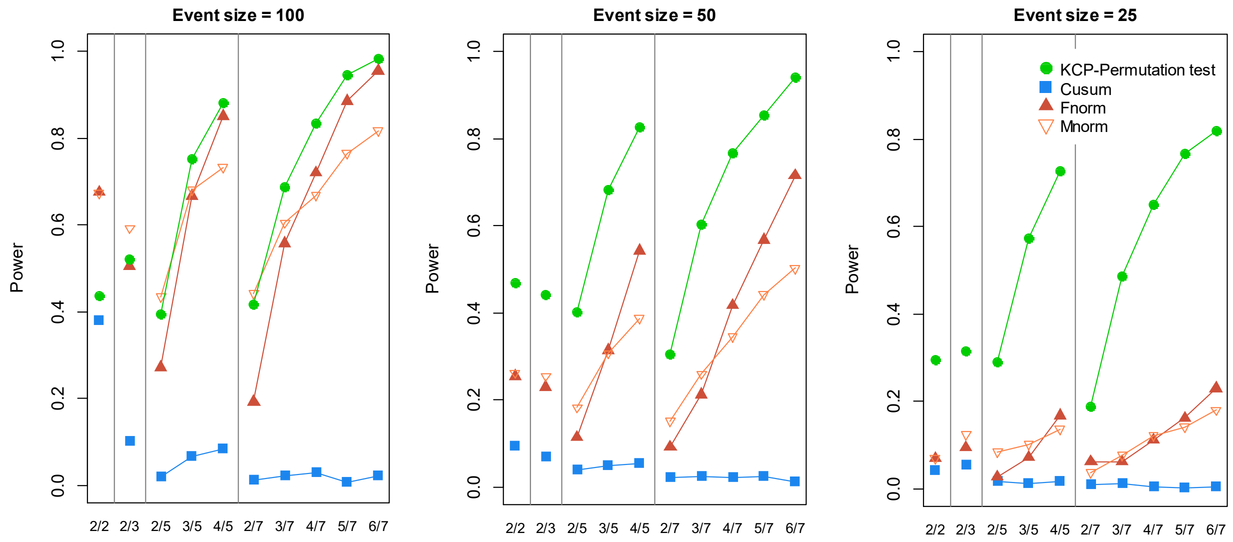

(a) (b) (c)

Figure S2. Power for settings with 4 correlation change points and varying event sizes: 100 (**a**), 50 (**b**) and 25 (**c**) time points. Power is averaged over all $\Delta\rho = 0.3, 0.5, 0.7$ and 0.9. In every panel, the power of the KCP permutation test (in green points), Cusum (blue squares), and the Frobenius norm (dark red filled upward triangles) and Maximum norm tests (light red empty downward triangles), were plotted across the simulation settings on the x-axis, where each setting is written as (no. of correlating variables *S*)/(total no. of variables *V*).

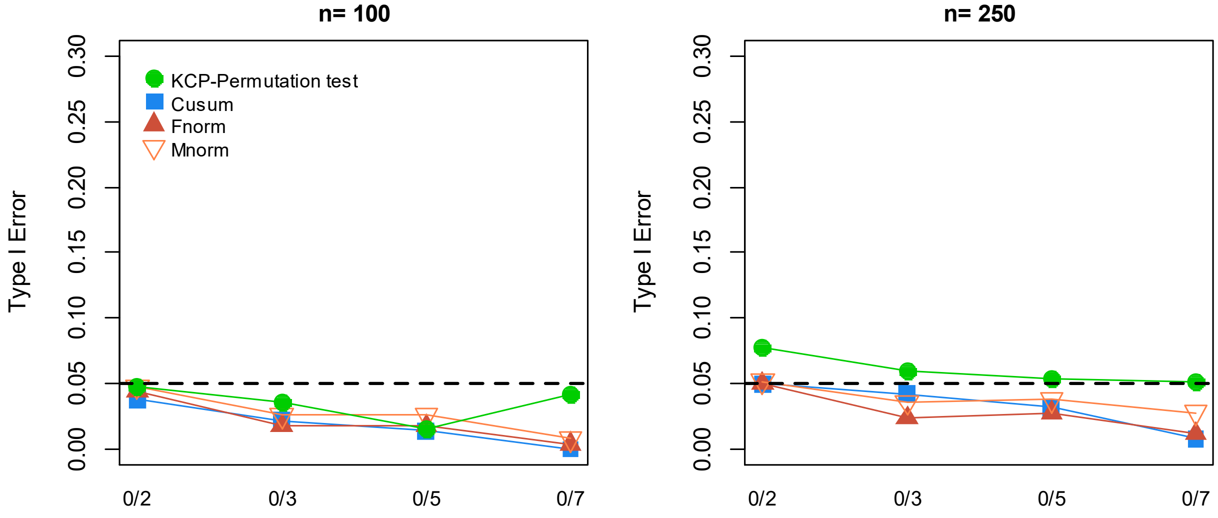

(a) (b)

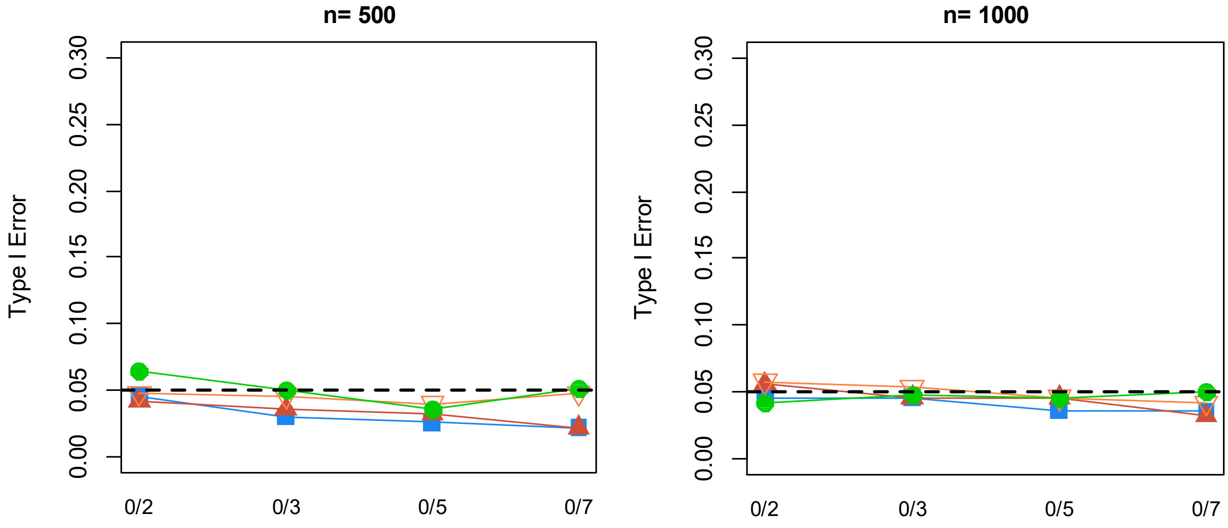

(c) **(d)**

Figure S3. Type I error for settings with no correlation change point. The four panels, (**a**), (**b**), (**c**) and (**d**), correspond to different sample sizes (*n*) tested: 100, 250, 500 and 1,000, respectively. In every panel, the false positive rate of the KCP permutation test (in green points), Cusum (blue squares), and the Frobenius norm (dark red filled upward triangles) and Maximum norm tests (light red empty downward triangles), were plotted across the simulation settings on the x-axis, where each setting is written as (no. of correlating variables *S*)/(total no. of variables *V*). The broken horizontal line indicates the nominal error rate (α=.05) set when implementing the methods.

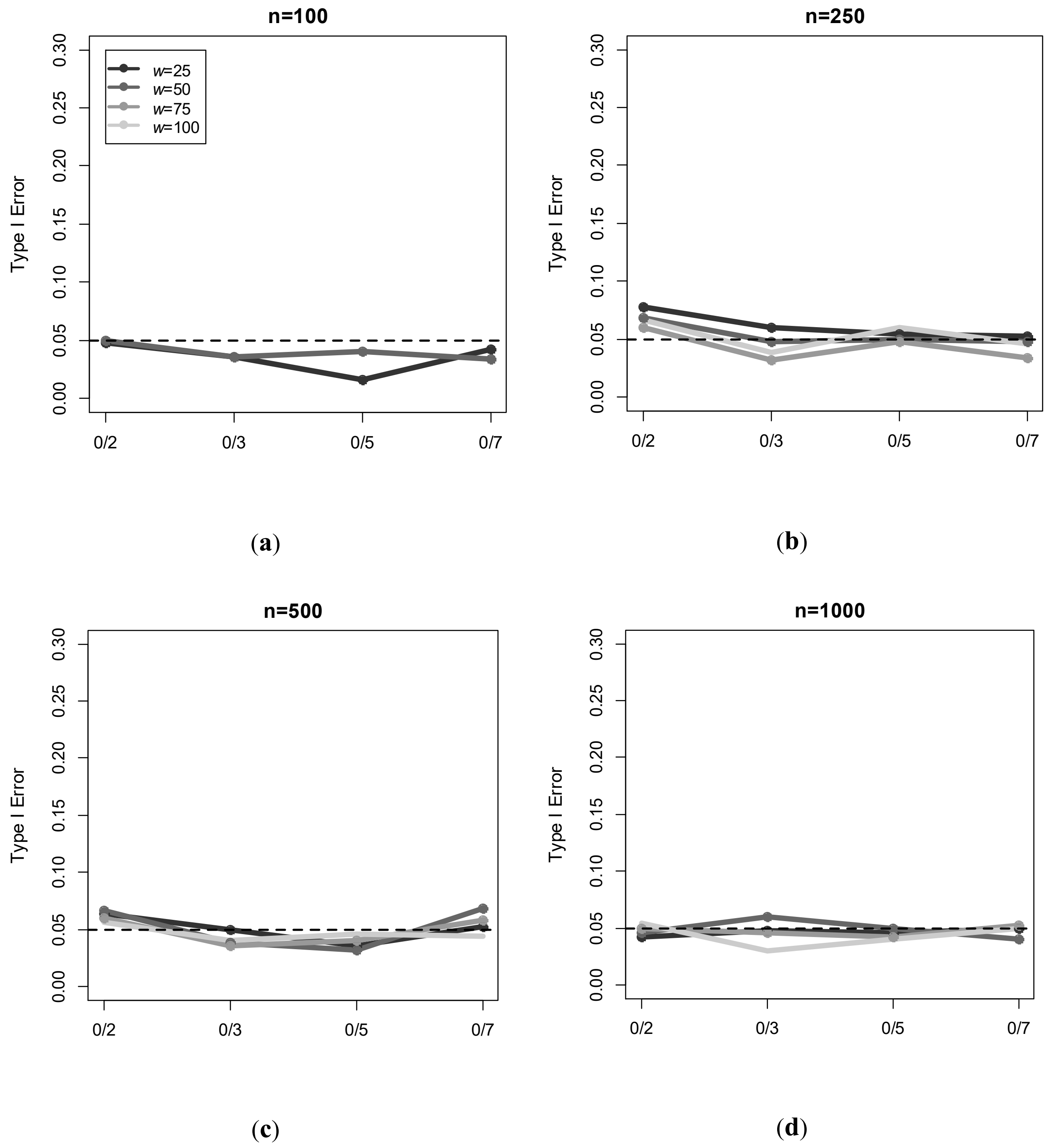

Figure S4. Type I error for KCP in settings with no correlation change point. The four panels, (**a**), (**b**), (**c**) and (**d**), correspond to different sample sizes (*n*) tested: 100, 250, 500 and 1,000, respectively. In every panel, the false positive rate of the KCP permutation test employing difference window sizes were plotted across the simulation settings on the x-axis, where each setting is written as (no. of correlating variables *S*)/(total no. of variables *V*). The

broken horizontal line indicates the nominal error rate (α=.05) set when implementing the methods. For *n*=100, *w*=75 and *w*=100 were not used as they lead to too few time points for the running correlations.

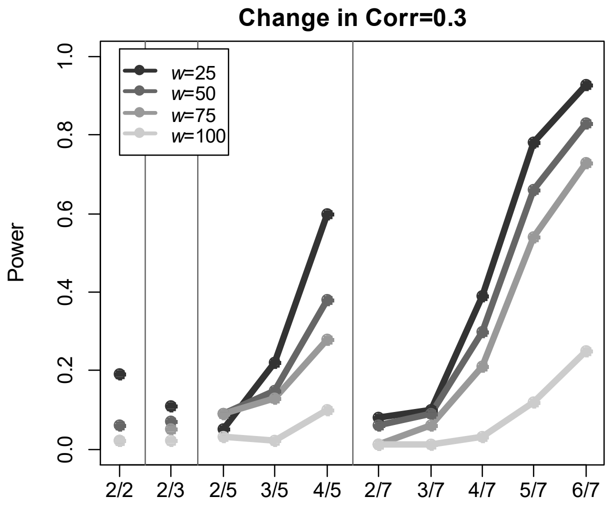

(**a**)

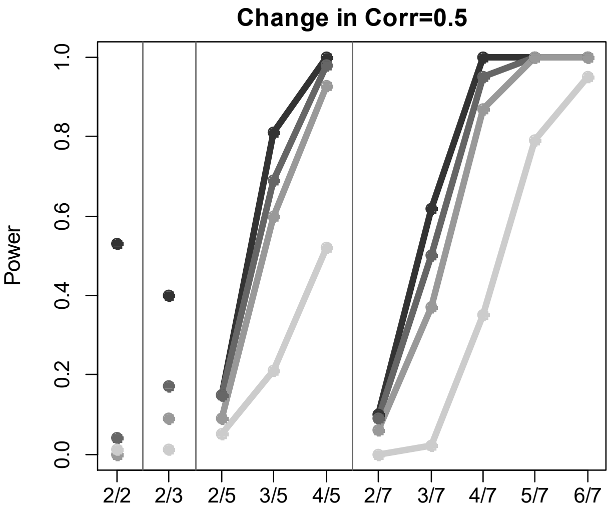

(**b**)

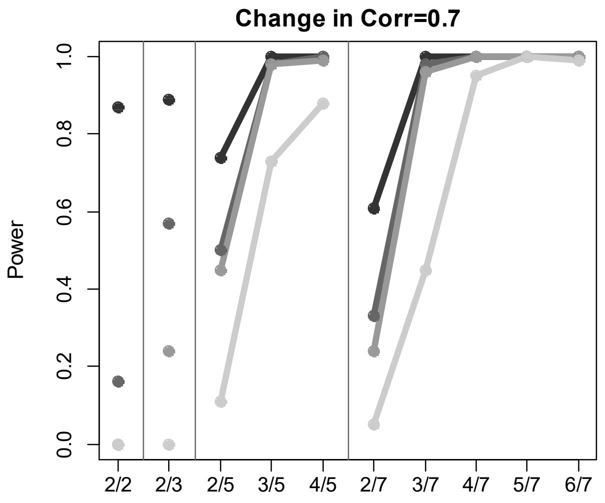

(**c**)

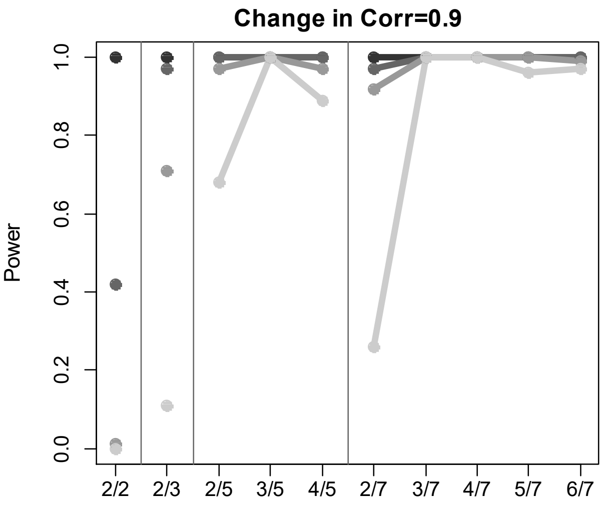

(**d**)

Figure S5. Power for KCP in settings with 2 correlation change points (event size = baseline size). The four panels, (**a**), (**b**), (**c**) and (**d**), correspond to the level of correlation change in the simulated data: 0.3, 0.5, 0.7 and 0.9, respectively. In every panel, the power of the KCP permutation test employing different window sizes were

plotted across the simulation settings on the x-axis, where each setting is written as (no. of correlating variables *S*)/(total no. of variables *V*).
